# Work-related factors predict changes in physical activity among nurses participating in a web-based worksite intervention: A randomized controlled trial

**DOI:** 10.1186/s12912-021-00739-4

**Published:** 2021-11-09

**Authors:** Jennifer Brunet, Melissa Black, Heather E. Tulloch, Andrew L. Pipe, Robert D. Reid, Jennifer L. Reed

**Affiliations:** 1 School of Human Kinetics, University of Ottawa, 125 University Private, Montpetit Hall, Room 339, Ottawa, ON K1N 6N5 Canada; 2 Institut du savoir de l’Hôpital Montfort (ISM), Hôpital Montfort, 713 Montreal Road, Ottawa, Ontario Canada; 3 Cancer Therapeutic Program, Ottawa Hospital Research Institute (OHRI), 725 Parkdale Avenue, Ottawa, Ontario Canada; 4 Division of Cardiac Prevention and Rehabilitation, University of Ottawa Heart Institute, 40 Ruskin Street, Ottawa, Ontario Canada; 5 Faculty of Medicine, University of Ottawa, 451 Smyth Road, Roger Guidon Hall, Ottawa, Ontario Canada

**Keywords:** Health promotion, Nursing, Randomized controlled trial, Workplace intervention, Physical activity

## Abstract

**Background:**

Despite the numerous benefits associated with physical activity (PA), most nurses are not active enough and few interventions have been developed to promote PA among nurses. A secondary analysis of raw data from a single-centre, three-arm parallel-group randomized controlled trial was conducted to assess whether work-related characteristics and general mood states predict changes in total weekly moderate-to-vigorous intensity PA (MVPA) and average daily step-count among nurses participating in a 6-week web-based worksite intervention.

**Methods:**

Seventy nurses (mean_age_: 46.1 ± 11.2 years) were randomized to an individual-, friend-, or team-based PA challenge. Participants completed questionnaires pre- and post-intervention assessing work-related characteristics (i.e., shift schedule and length, number of hours worked per week, work role) and general mood states (i.e., tension, depression, anger, confusion, fatigue, vigour). Participants received a PA monitor to wear before and during the 6-week PA challenge, which was used to assess total weekly MVPA minutes and average daily step-count. Data were analyzed descriptively and using multilevel modeling for repeated measures.

**Results:**

Change in total weekly MVPA minutes, but not change in average daily step-count, was predicted by shift schedule (rotating vs. fixed) by time (estimate = − 17.43, SE = 6.18, *p =* .006), and work role (clinical-only vs. other) by time (estimate = 18.98, SE = 6.51, *p =* .005). General mood states did not predict change in MVPA or change in average daily step-count.

**Conclusions:**

Given that nurses who work rotating shifts and perform clinical work showed smaller improvements in MVPA, it may be necessary to consider work-related factors/barriers (e.g., time constraints, fatigue) and collaborate with nurses when designing and implementing MVPA interventions in the workplace.

**Trial registration:**

ClinicalTrials.gov: NCT04524572. August 24, 2020. This trial was registered retrospectively. This study adheres to the CONSORT 2010 statement guidelines.

**Supplementary Information:**

The online version contains supplementary material available at 10.1186/s12912-021-00739-4.

## Background

Although nurses play important and valued roles in society, they do so at considerable cost to their physical and mental health. Studies indicate that many nurses have poor health outcomes and risk factors, including excess weight, high cholesterol, hypertension, diabetes, anxiety, depression, back problems, and arthritis [1–3]. Many investigations also suggest that nurses experience poor mental health [1, 3, 4], with several studies showing elevated levels of depression, emotional distress, and stress among nurses [2, 5–7]. Poor physical and mental health may lead nurses to be absent from work. A national survey found that 61% of nurses working in Canada took time off work for health reasons [3], and more than 24,000 (9%) were absent each week [8]. Experts estimate that health-related absenteeism among nurses costs the Canadian healthcare system nearly 1 billion dollars annually [8]. Yet, most healthcare efforts are focused on patient-care, and nurses who play a pivotal role in providing such care, receive limited support to prevent and manage the potential negative health consequences of nursing.

There is clear evidence that regular physical activity (PA) is associated with improved physical and mental health in the general population [9, 10]. Regular PA is also associated with improved work capacity and lower rates of employee burnout among healthcare workers [11, 12]. Although nurses’ personal health and workplace engagement could be supported and improved by regular PA, many report engaging in little to no PA [2, 13–15]. A commonly accepted and converging viewpoint is that promoting regular PA among nurses may encourage nurses to promote regular PA among their patients while also helping to enhance their own physical and mental health [16].

Several systematic reviews indicate that a variety of interventions (e.g., supervised exercise programs, behavioural support, PA counselling) designed to promote PA behaviour in the general population can be effective at increasing PA levels [17–19]. However, traditional interventions require participants to attend scheduled PA sessions at a facility, which may not be feasible or acceptable for nurses who work long shifts (e.g., 12 h) and/or rotating shifts (i.e., working a combination of days, evenings, and/or nights). The demanding characteristics of nursing roles may help to explain why, of the few interventions designed to promote PA behaviour among nurses, most were ineffective at increasing PA levels [20]. Web-based interventions delivered in the workplace may be particularly beneficial as they offer simple and flexible approaches to promoting PA behaviour by helping to address common barriers (e.g., lack of time, fatigue) nurses face when attempting to participate in PA.

There remains a need to better understand how work-related characteristics associated with nursing roles (e.g., long shifts, rotating shifts, high total hours per week, and clinical responsibilities) might impact nurses’ responses to interventions delivered in the workplace. While performing clinical duties, nurses are often highly physically and emotionally involved in providing care to their patients and perceive their work as highly taxing [7, 21]. These nurses, in turn, may be less likely to make changes to their PA behaviour especially if they lack confidence that they will have sufficient time or energy to make such changes. Nurses who report higher levels of stress also engage less in self-care behaviours, including PA [22]. Additionally, low energy may be a barrier to PA change in nurses who work longer shifts or rotate between working days, evenings, and nights. These characteristics have been associated with lower PA levels [2]. Research examining the assertion that nurses with more taxing work-related demands are less likely to change their PA behaviour would provide valuable insights to inform the development of interventions tailored to the needs of these potentially at-risk nurses. If work-related demands are, in part, the reason that nurses respond poorly to PA interventions delivered in the workplace, such knowledge will help to identify nurses who face specific challenges in changing their PA behaviour. Strategies and modes of intervention may need to differ according to the varying shift lengths, shift schedules, work hours, and work responsibilities of particular nurses.

It is important, however, to not limit the analysis of predictors of PA change solely to work-related characteristics as these may be difficult to change. There is growing research interest in the relationships of affect and mood with PA behaviour. It has been shown that affective states (e.g., pleasure/displeasure, tension/relaxation, sluggishness/excitement, depression) predict future PA behaviour within the general population [23, 24]. Although the importance of PA-induced changes in affect should not be overlooked, whether general affective states predict PA behaviour has received relatively little attention, and most researchers have considered affect as an outcome of PA. In the few studies that have examined general affective states as predictors of PA behaviour [25–27], affective states were found to predict PA behaviour. Emerson and Dunsiger [26] showed that those experiencing a positive mood unrelated to PA participation were more likely to be active later that same day. These findings support observations that individuals may be more likely to engage in health promoting behaviours when their mood is more positive [see 24, 28]. As such, it is possible that positive affective states may also predict nurses’ response to a workplace PA intervention whereby nurses with greater mood disturbances (i.e., those who feel tense, depressed, angry, confused, and fatigued, and who are lacking vigour) may be less likely to change their PA behaviour.

### Study aims

Whilst studies examining correlates of PA behaviour among nurses have been published [e.g., 2, 29], studies specifically examining predictors of PA behaviour among nurses who have been recruited and enrolled into a workplace intervention are sparse. Considering that worksite interventions have been shown to be effective at promoting PA behaviour [30, 31], studies need to move beyond testing intervention effectiveness to examine confounding factors that may increase or decrease intervention effectiveness (e.g., work-related characteristics, mood states). The current study is a secondary analysis of raw data from a parallel-randomized controlled trial (RCT) assessing the impact of a web-based worksite intervention on PA behaviour and cardiovascular health among nurses working in a tertiary-care cardiovascular institute [32]. A web-based worksite intervention strategy was chosen to address common barriers to PA, including lack of time and fatigue [33, 34], that may be exacerbated by the long hours and irregular shifts of nurses [35]. The purpose of this study was to assess whether work-related characteristics (i.e., number of hours worked per week, shift schedule, shift length, work role) and mood states (i.e., tension, depression, anger, confusion, fatigue, vigour) predicted changes in total weekly moderate-to-vigorous intensity PA (MVPA) and average daily step-count among nurses who participated in a 6-week web-based worksite intervention. We hypothesized that certain job characteristics, namely working non-clinical roles, fewer hours, a fixed shift schedule, and shorter shifts, and having better general mood states would predict greater increases in MVPA minutes and step-count during the intervention.

## Methods

### Study design

This study was a single-centre, parallel-group, RCT designed to compare the effects of three interventions on PA behaviour and cardiovascular health indicators. The study protocol was approved by the University of Ottawa Heart Institute Research Ethics Board (Protocol No. 20130429) and was retrospectively registered in the ClinicalTrials.gov database (No. NCT04524572) on August 24, 2020. All participants provided written informed consent prior to participating in any study-related activities. The Consolidated Standards of Reporting Trials (CONSORT) 2010 Statement (http://www.consort-statement.org/consort-2010) was followed while writing this manuscript, and the completed CONSORT checklist can be found in Supplemental File 1.

### Participants

Registered nurses were recruited from September to November 2013 via posters distributed throughout the University of Ottawa Heart Institute, and announcements during nurse-attended meetings and morning rounds. Nurses who were non-ambulatory, unwilling to wear an accelerometer, did not have access to the Internet, and/or were unable (or unwilling) to provide written informed consent were excluded. Also, nurses who were pregnant or lactating, unable to read and understand English, had medical contraindications to exercise, and/or already using an activity monitor to track their PA levels were not eligible to participate in the trial.

### Assessments and procedures

Participants met with research staff to provide written informed consent, complete baseline self-report measures, and have their resting blood pressure, resting heart rate, and anthropometric measurements recorded. At this time, participants received a Tractivity® activity monitor (Tractivity®, Vancouver, BC) and were then randomized to one of the three intervention groups. Following the 6-week intervention phase, participants met with research staff, who were blinded to participants’ group assignment, to complete follow-up self-report measures and have their resting blood pressure, resting heart rate, and anthropometric measurements recorded. These assessments are described in detail elsewhere [see 32 ] .

Work-related characteristics were assessed at baseline by having participants self-report average length of shifts worked, shift schedule (day, night, rotating), number of hours worked per week, and their role (clinical, research, clinical plus research, managerial, managerial plus clinical). For the analyses, dummy variables were created for variables with more than two categorical response options as in past studies [2]. Shift sch edule was dichotomized as 0 = fixed day or fixed night shifts vs. 1 = rotating shifts. Role was dichotomized as 0 = clinical-only vs. 1 = any combination of clinical, managerial, and research to reflect typical area-specific workload (i.e., nurse-to-patient ratio) and type of work performed in these roles. Shift length was dichotomized as 0 = primarily shifts of 8 h vs. 1  = primarily shifts of 12 h.

General mood states were assessed at baseline by having participants complete the Profile of Mood State s [PO MS; 36]. The POMS is a 65-item scale that assesses six different mood states (i.e., tension, depression, anger, confusion, fatigue, vigo ur) over the previous 7 days using various adjectives, which participants rated on a 5-point Likert scale ranging from 0 (“*not at all*”) to 4 (“*extremely*”). POMS scores have goo d internal reliability and construct validity [36, 37]. Total mood disturbance and s ubscale scores (i.e., tension, depression, anger, fatigue, confusion, vigour) were analyzed as continuous variables and examined separately as predictors of total weekly MVPA minutes and average daily step-count.

 The outcome variables of interest (i.e., total weekly MVPA minutes and average daily step-count) were assessed usin g a Tractivity® activity monitor. All participants were asked to wear the device on a continuous basis (i.e., daily from waking until bedtime and to remove it only when bathing or engaging in water-related activities) 1 week before participating in the intervention (‘baseline phase’ assessment) and throughout the 6-week interve ntion period (‘intervention phase’ assessment). Activity monitors were calibrated for stride length prior to the baseline assessment [see 32 for futher details]. Participants uploaded their activity data at times and frequencies of their choosing t hroughout the intervention phase, and research staff uploaded participants’ activity data into an online Tractivity® program prior to and at the end of the intervention phase.

The Tractivity® activity monitor data was used to calculate total weekly MVPA minutes and average daily step-count. First, downloaded data were screened to identify valid days. Data were considered valid and were included in the analysis if wear time was at least 10 h for any given day [38]. Next, using activity monitor determined step-counts for valid wear days, total weekly MVPA minutes and average daily step-count were computed for the baseline phase and each week of the 6-week intervention. An established threshold-value of 100 steps per minute was used to identify minutes of MVPA [39, 40]. For the purposes of this study, only bouts of MVPA lasting at least 10 minutes were included when calculating total daily weekly MVPA minutes [41, 42].

### Intervention groups and randomization

Participants were randomized to one of three groups (i.e., individual-, friend-, or team-based PA challenge) by research staff in a 1:1:1 ratio using the “RAND” function of a software spreadsheet program (Excel, Microsoft, Washington, USA). Participants were then notified of their group assignment via email and had a Tractivity® web account created for them when they received their monitor. Those randomized to the individual challenge group were able to log onto their Tractivity® web account at any time during the intervention phase to track their own PA behaviour. Nurses in the friend challenge group were also able to log onto their Tractivity® web account at any time during the intervention phase to track their own PA behaviour, but they could also monitor the PA behaviour of one other anonymous participant. Nurses in the team challenge group were assigned to one of five teams; they were able to monitor their own PA behaviour and compare the average PA behaviour of their team to the average of the other four teams. The main difference across the groups was thus the group size/composition with whom participants could compare their PA behaviour with.

### Sample size

A post-hoc power analysis revealed that a sample size of 76 participants provides adequate power (1–β = .92) to detect significant differences in PA within and between groups of small magnitude (i.e., eta-squared value of .022 with an alpha of .05).

### Data analysis

#### Preliminary analyses

Data were analyzed using SPSS (version 25; IBM Corp, Armonk, NY, USA). Initially, descriptive statistics were computed for the principal study variables. The data for total weekly MVPA minutes, average daily step-count, and baseline POMS scores (i.e., tension, depression, anger, fatigue, confusion, and total mood disturbance) were not normally distributed. A two-step approach was applied to normalize the data [see 32 for futher details].

Next, two sets of multilevel models for repeated measures were estimated. First, unconditional multilevel growth models were estimated to summarize how total weekly MVPA minutes and average daily step-count changed over time. Time was expressed in a linear form (time) in an initial model and compared to an alternate model where time was expressed in a linear (time) and quadratic form (time squared). Models were estimated using Restricted Maximum Likelihood (REML) with an *unstructured covariance* error structure, which considers that no two pairs of observations are equally correlated. Models were then compared using the Aikaike’s Information Criterion (AIC [43]) and the Bayesian Information Criterion (BIC [44]) to identify which form of change (i.e., linear or quadratic) gave the best model fit. Based on the AIC and BIC values, the optimal form retained for both outcomes was quadradic, suggesting change was nonlinear (see results below). Therefore, linear and quadratic terms of time were included in all subsequent models. Then, the fixed effects of group, group by time, and group by time squared were entered as predictors of total weekly MVPA minutes and average daily step-count. Both models revealed that group was not significantly related to initial levels or rate of change in total weekly MVPA minutes and average daily step-count (see results below), meaning there was no significant differences between the groups at baseline or in terms of change throughout the intervention. Therefore, group factors were not included in subsequent models.

#### Main analyses

The hypothesized predictive associations were examined by testing conditional multilevel models to account for the hierarchical structure of the data – repeated measurements nested within participants. A series of models were tested to explore systematic inter-individual differences in the intercept (representing baseline total weekly MVPA minutes and average daily step-count) and slopes (representing rate of change in total weekly MVPA minutes and average daily step-count). Specifically, POMS scores and work-related characteristics at baseline and their interaction with growth parameters (i.e., time and time squared) were entered as predictors of MVPA and step-count. Owing to limitations in sample size, each predictor and its interactions with growth parameters were tested in separate models to retain power.

## Results

**Fig. 1:**
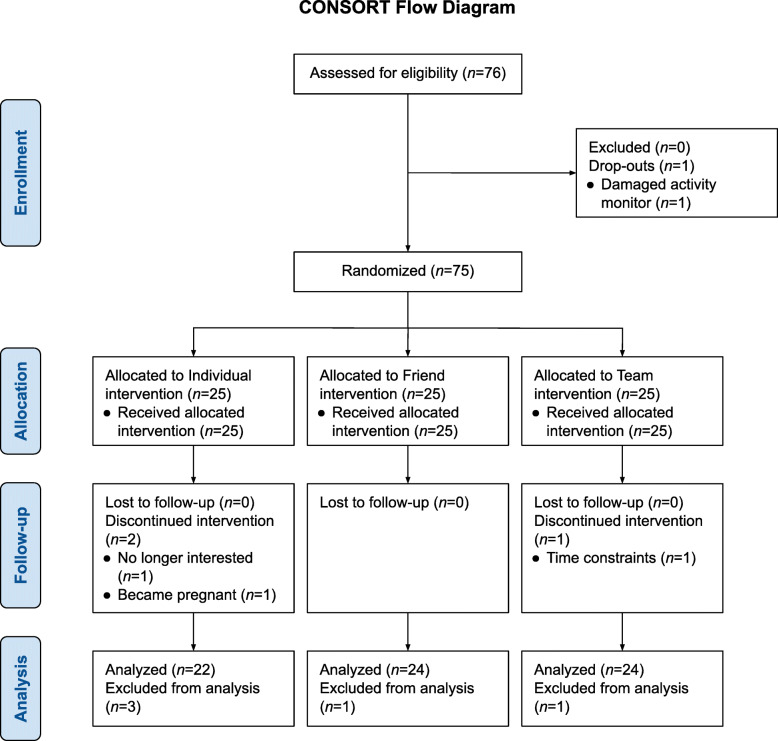
Consolidated Standards of Reporting Trials (CONSORT) flow diagram of nurses recruited and reasons for withdrawals

In total, 76 nurses were recruited and 75 were randomized (see Fig. 1 for CONSORT flow chart). For this study, data from 70 participants (92%) who completed the 6-week intervention, and for whom data for all predictor variables of interest at baseline was available, were analyzed. Baseline characteristics of the analytical sample are provided in Table 1 and characteristics of the full sample are available elsewhere [see 32]. In summary, participants were primarily female (97.1%), between the ages of 22 and 65 years (46.0 ± 11.2 years), and had a mean body mass index of 27.3 ± 5.5 kg/m^2^. Most worke d day shifts (52.2%) in clinical positions without additional research or management responsibilities (69.6%).

**Table 1:** Participant characteristics at baseline (*n* = 70)

Characteristics	Values
Demographics [mean (SD)]	
Age [mean years (SD)]	46.1 (11.2)
Body mass index [mean kg/m^2^ (SD)]	27.3 (5.5)
Female [*n* (%)]	68 (97.1)
Work role [*n* (%)]	
Clinical	48 (69.6)
Research	3 (4.3)
Research + clinical	3 (4.3)
Managerial	9 (13.0)
Managerial + clinical	6 (8.7)
Shift schedule [*n* (%)]	
Days only (fixed)	37 (52.9)
Nights only (fixed)	5 (7.1)
Rotating	28 (40.0)
Shift length [*n* (%)]	
8 h	30 (42.9)
12 h	32 (45.7)
Mixed	8 (11.4)
Hours worked per week [mean (SD)]	36.2 (8.6)
Baseline mood states [mean (SD)]	
Tension	7.4 (5.7)
Depression	6.2 (7.6)
Anger	5.8 (6.6)
Fatigue	7.7 (5.5)
Confusion	4.8 (4.0)
Vigour	15.0 (5.6)
Total mood disturbance	16.9 (30.0)
Total weekly moderate-to-vigorous intensity physical activity minutes [mean (SD)]	
Baseline (Week 0)	29.0 (50.0)
Post-intervention (Week 6)	24.2 (43.1)
Average daily step-count [mean (SD)]	
Baseline (Week 0)	9062.9 (3499.9)
Post-intervention (Week 6)	6679.2 (5472.0)

The fixed estimates of our unconditional multilevel growth models indicated that there was a significant intercept (estimate = 35.84, SE = 4.63, *p* < .001) and linear and quadratic slopes for total weekly MVPA minutes (linear estimate = 10.72, SE = 3.19, *p* = .001; quadradic estimate = − 2.16, SE = 0.53, *p* < .001), indicating change over time in a curvilinear fashion, whereby an initial increase in MVPA minutes was followed by a decrease. In addition, the random effects indicated that there was significant inter-individual variability in MVPA minutes at baseline (*p* < .001), and in the linear (*p = .*045) and quadratic (*p =* .032) rates of change over time (i.e., slopes). Analysis of daily step-count revealed a similar pattern. The intercept (estimate = 8858.14, SE = 378.17, *p <* .001) and the linear and quadratic slopes (linear estimate = 710.84, SE = 271.32, *p* = .011; quadratic estimate = − 166.49, SE = 45.48, *p =* .001) were significant. The random effects of step-count indicated that there was significant inter-individual variability in the intercept (*p* = .002) and in the linear (*p* = .013) and quadratic slopes (*p* = .006).

**Fig. 2:**
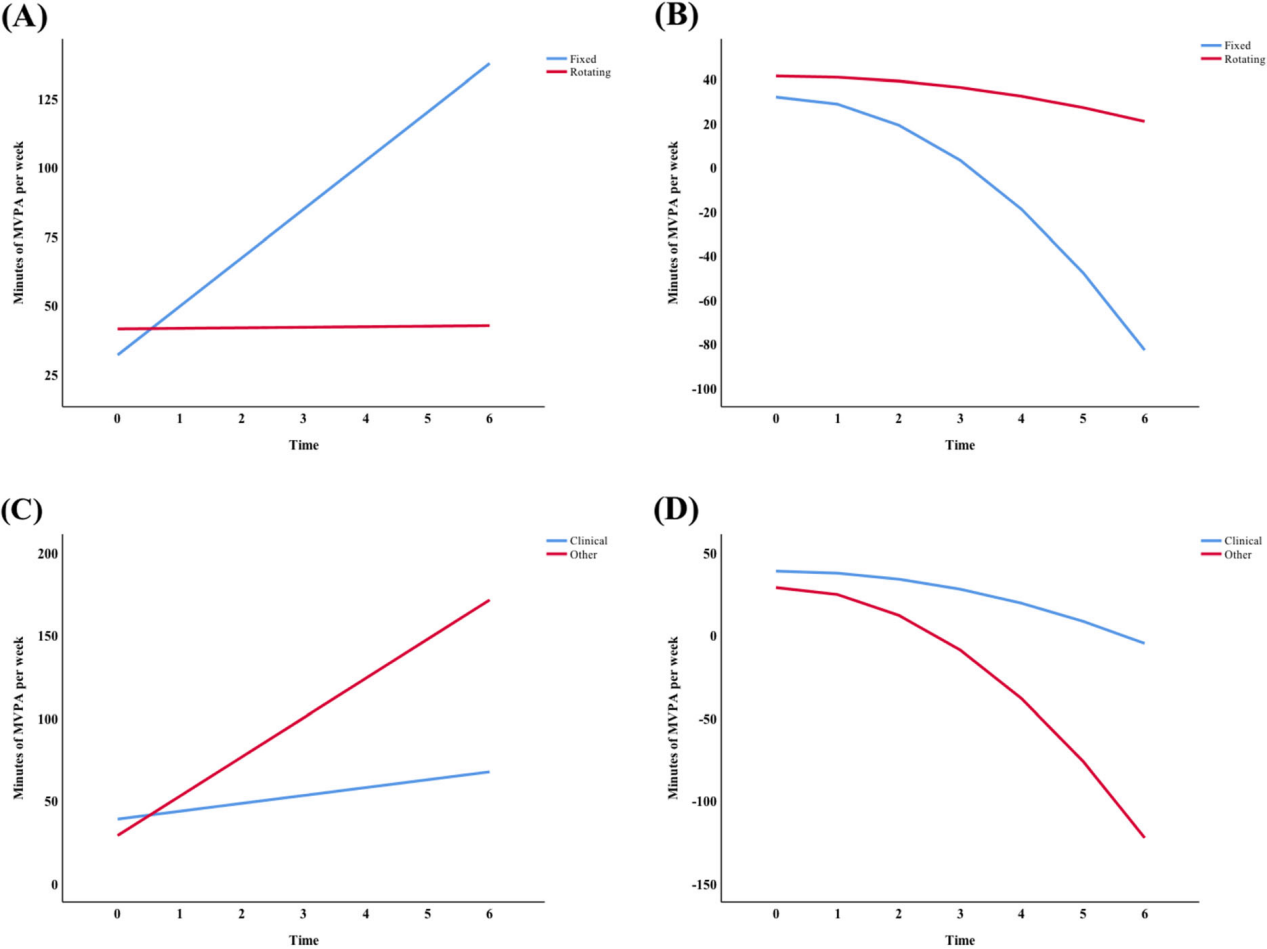
Interaction of (**A**) work role and time, (**B**) work role and time squared, (**C**) shift schedule and time, and (**D**) shift schedule and time squared in predicting total weekly MVPA minutes

In comparison to the unconditional multilevel models, the conditional multilevel models with significant predictors resulted in a reduction in − 2 Restricted Log Likelihood, AIC, and BIC values, indicating better fit and suggesting that addition of these predictors helped to explain significant inter-individual differences in total weekly MVPA minutes and daily step-count. Table 2 contains the fixed effects associated with work-related predictors, whereas Table 3 contai ns the fixed effects associated with mood states predictors. Overall, we found that work role and shift schedule predicted change in total weekly MVPA minutes but not change in average daily step-count, and that the mood states did not predict change in either outcome. Specifically, the interactions between work role and time (estimate = 18.98, SE = 6.51, *p =* .005) and between work role and time squared (estimate = − 2.99, SE = 1.07, *p =* .007) were significant. To understand the nature of these associations, the interactions were probed by tests of simple slopes at specific values where 0 = performing exclusively clinical duties and 1 = performing managerial or research responsibilities [see 45, 46]. Probing revealed that nurses performing managerial or research responsibilities demonstrated greater increases in their total weekly MVPA minutes initially than nurses performing exclusively clinical duties (Fig. 2a); however, the rate of change declined less rapidly toward the end of the intervention phase for nurses performing exclusively clinical duties (Fig. 2b). In addition, the interactions between shift schedule and time (estimate = − 17.43, SE = 6.18, *p =* .006) and between shift schedule and time squared (estimate = 2.62, SE = 1.02, *p* = .013) were significant. Probing showed that nurses working fixed shifts demonstrated significantly greater increases in their total weekly MVPA minutes initially than nurses working rotating shifts ( Fig. 2c); however, the rate of change declined less rapidly toward the end of the intervention phase for nurses working rotating shifts (Fig. 2d). None of the mood sta te scores(i. e., POMS total mood disturbance, POMS subscale scores) were associated with initial levels or rates of change in total weekly MVPA minutes.

**Table 2:** Fixed effects and fit statistics for multilevel growth models with work-related characteristics as predictors

	Model 1	Model 2	Model 3	Model 4
**Total weekly MVPA minutes**				
Intercept	35.85 (4.64)^†^	34.24 (6.87)^†^	32.02 (6.02)^†^	38.92 (5.61)^†^
Time	10.76 (3.21)**	16.46 (4.60)**	17.63 (3.89)^†^	4.77 (3.64)
Time squared	− 2.16 (0.53)^†^	−3.08 (0.76)^†^	−3.19 (0.64)^†^	−1.21 (0.60)*
Hours	−0.44 (0.54)			
Hours*time	−0.08 (0.39)			
Hours*time squared	0.02 (0.06)			
Shift length		2.89 (9.36)		
Shift length*time		−10.80 (6.31)		
Shift length*time squared		1.75 (1.05)		
Shift schedule			9.48 (9.59)	
Shift schedule*time			−17.43 (6.18)**	
Shift schedule*time squared			2.62 (1.02)*	
Role				−9.94 (10.11)
Role*time				18.98 (6.51)**
Role*time squared				−2.99 (1.07)**
**Goodness of fit**				
-2LL	4700.008	4681.183	4676.194	4676.361
AIC	4714.008	4695.183	4690.194	4690.361
BIC	4742.987	4724.162	4719.173	4719.340
**Average daily step-count**				
Intercept	8853.97 (379.25)^†^	8435.45 (550.29)^†^	8594.26 (482.24)^†^	9264.44 (449.61)^†^
Time	714.68 (273.37)*	796.15 (395.87)*	691.27 (349.16)	497.86 (326.38)
Time squared	−166.94 (45.84)**	−183.46 (66.32)**	− 154.38 (58.50)**	− 128.40 (54.65)*
Hours	−35.03 (44.43)			
Hours*time	−5.60 (32.82)			
Hours*time squared	0.20 (5.51)			
Shift length		799.73 (756.92)		
Shift length*time		− 160.21 (547.04)		
Shift length*time squared		32.07 (91.68)		
Shift schedule			688.86 (779.17)	
Shift schedule*time			47.44 (561.28)	
Shift schedule*time squared			−30.77 (94.00)	
Role				− 1313.1824 (808.30)
Role*time				684.99 (583.72)
Role*time squared				− 122.26 (97.69)
**Goodness of fit**				
-2LL	8830.008	8813.162	8812.931	8810.698
AIC	8844.008	8827.162	9926.931	8824.698
BIC	8872.988	8856.142	8855.911	8853.677

* MVPA* moderate-to-vigorous intensity physical activity, *Hours* hours worked per week, *−2LL* −2 Restricted Log Likelihood, *AIC* Aikaike’s Information Criterion, *BIC* Bayesian Information Criterion

*p < .05, **p < .01, ^†^*p* < .001

**Table 3:** Fixed effects and fit statistics for multilevel growth models with mood states as predictors

	Model 1	Model 2	Model 3	Model 4	Model 5	Model 6	Model 7
**Total weekly MVPA minutes**							
Intercept	36.22 (4.69)^†^	36.33 (4.71)^†^	36.29 (4.71)^†^	36.07 (4.73)^†^	36.17 (4.67)^†^	36.42 (4.69)^†^	36.29 (4.71)^†^
Time	10.96 (3.26)**	10.98 (3.25)**	10.99 (3.23)**	10.92 (3.24)**	11.05 (3.25)**	10.76 (3.24)**	10.98 (3.25)**
Time squared	−2.20 (0.54)^†^	− 2.20 (0.54)^†^	− 2.21 (0.53)^†^	− 2.19 (0.53)^†^	−2.21 (0.54)^†^	− 2.17 (0.53)^†^	−2.20 (0.54)^†^
Tension	0.65 (0.90)						
Tension*time	−0.11 (0.63)						
Tension*time squared	−0.00 (0.10)						
Depression		−0.01 (0.69)					
Depression*time		−0.14 (0.48)					
Depression*time squared		0.01 (0.08)					
Anger			0.21 (0.77)				
Anger*time			−0.54 (0.53)				
Anger*time squared			0.09 (0.09)				
Fatigue				0.24 (0.92)			
Fatigue*time				0.44 (0.63)			
Fatigue*time squared				−0.09 (0.10)			
Confusion					1.40 (1.27)		
Confusion*time					−5.56 (0.90)		
Confusion*time squared					0.07 (0.15)		
Vigour						0.53 (0.88)	
Vigour*time						0.59 (0.60)	
Vigour*time squared						−0.09 (0.10)	
TMD							0.04 (0.17)
TMD*time							−0.03 (0.12)
TMD*time squared							0.00 (0.19)
**Goodness of fit**							
-2LL	4532.575	4634.343	4633.189	4630.450	4629.813	4628.514	4642.996
AIC	4646.575	4648.343	4647.189	4644.450	4643.813	4642.514	4656.996
BIC	4675.448	4677.216	4676.062	4673.323	4672.686	4671.387	4685.869
**Average daily step-count**							
Intercept	8842.10 (386.43)^†^	8848.16 (384.23)^†^	8845.54 (386.53)^†^	8854.70 (386.82)^†^	8843.07 (385.37)^†^	8911.60 (365.04)^†^	8849.25 (384.58)^†^
Time	734.24 (276.17)*	737.43 (275.47)**	739.45 (275.67)**	732.88 (273.55)**	733.87 (275.92)*	690.32 (276.58)*	735.00 (275.07)*
Time squared	− 170.16 (46.23)^†^	−170.58 (45.98)^†^	− 171.44 (46.25)^†^	− 167.20 (45.89)^†^	−170.02 (46.11)^†^	− 161.99 (46.20)**	−170.14 (45.90)^†^
Tension	22.05 (73.71)						
Tension*time	16.12 (53.38)						
Tension*time squared	−4.30 (8.95)						
Depression		−50.56 (56.46)					
Depression*time		22.41 (40.69)					
Depression*time squared		−6.17 (6.79)					
Anger			8.80 (63.36)				
Anger*time			−18.48 (45.05)				
Anger*time squared			1.35 (7.55)				
Fatigue				−7.12 (74.84)			
Fatigue*time				56.34 (52.81)			
Fatigue*time squared				−11.45 (8.86)			
Confusion					66.72 (105.10)		
Confusion*time					27.84 (76.41)		
Confusion*time squared					−8.97 (12.78)		
Vigour						183.97 (68.17)^†^	
Vigour*time						−14.74 (51.50)	
Vigour*time squared						5.67 (8.60)	
TMD							−11.23 (13.55)
TMD*time							7.01 (9.76)
TMD*time squared							−1.69 (1.63)
**Goodness of fit**							
-2LL	8701.030	8699.874	8701.851	8694.425	8697.434	8684.503	8708.974
AIC	8715.030	8713.874	8715.951	8708.425	8711.434	8598.503	8722.974
BIC	8743.902	8742.747	8744.724	8737.298	8740.307	8727.375	8751.847

*MVPA* moderate-to-vigorous intensity physical activity, *TMD* total mood disturbances, *−2LL* −2 Restricted Log Likelihood, *AIC* Aikaike’s Information Criterion, *BIC* Bayesian Information Criterion

*p < .05, **p < .01, ^†^*p* < .001

Similar to total weekly MVPA minutes, none of the mood states scores (i.e., POMS total mood disturbance, POMS subscale scores) were associated with initial levels or rates of change in average daily step-count, with one exception (Table 3). The fixed effect of baseline vigour on step-count was significant (estimate = 183.97, SE = 68.17, *p* = .009), indicating that nurses who reported higher levels of vigour at baseline had higher daily step-count in general. The interactions between vigour and time and between vigour and time squared were not significant, indicating that baseline levels of vigour did not impact the rate of change in daily step-count. None of the work-related characteristics predicted initial step-count or change in step-count as none had significant main or interactive effects, respectively.

## Discussion

Strong evidence suggests that low levels of PA increases the likelihood of poor physical and mental health and diminished wellbeing [10, 11]. Many nurses do not meet current PA guidelines [3]. Thus, efforts to promote regular PA among nurses are warranted. We delivered a web-based worksite intervention and previously demonstrated that it was effective at increasing nurses’ total weekly MVPA minutes and average daily step-count; however, the effects of the intervention were short-lived [32]. In this article, analysis using multilevel modelling allowed us to detect significant variability between nurses' responses to the intervention and, in turn, to explore predictors that may explain some of this variability. On the basis of previous research involving nurses [22, 23], we hypothesized that certain job characteristics (i.e., non-clinical role, fewer hours worked per week, a fixed shift schedule, shorter shift length) and better general mood states would predict greater increases in MVPA minutes and step-count during the intervention. We found that work r ole and shift schedule predicted change in total weekly MVPA minutes but not change in average daily step-count, and that the mood states did not predict change in either outcome. These findings enrich the conclusions derived in Reed et al. [32] and suggest several considerations for developing and tailoring interventions to promote PA, reflecting the diverse needs of nurses.

Consistent with previous cross-sectional work [3, 16], certain work-related characteristics (i.e., r ole and shift schedule) predicted nurses’ responses to our intervention in terms of their MVPA behaviour. These findings highlight that nurses are a heterogenous population, and that interventions designed to promote PA behaviour may differentially affect nurses’ MVPA behaviour based on their working environments. Nurses working in roles with managerial or research responsibilities and those working a fixed shift schedule showed a curvilinear change trajectory, whereby an initial increase in total weekly MVPA minutes was followed by a progressive decrease. In contrast, nurses working in clinical roles and rotating shifts showed little to no change in total weekly MVPA minutes in response to the intervention. The lack of effect of the intervention on total weekly MVPA minutes among such nurses may be attributable to the inherent challenges and pressures associated with these roles. To speculate, nurses working in clinical roles are more involved in-patient care, may have comparatively more physically demanding work, higher levels of light intensity occupational PA [47], and less flexibility to perform self-care activities during their shifts in comparison to nurses performing managerial or research tasks. These factors may contribute to physical health issues (e.g., back pain or musculoskeletal disorders [48]), and to stress or emotional distress [8, 49], which could make it more difficult for them to increase their participation in MVPA both at work and across other settings. Nurses working rotating schedules may similarly face constraints that could lead to lower PA levels and poorer response to PA interventions. Irregular shift work can lead to feelings of time scarcity and constraints, social disruption, and psychological distress [50–52]. Nurses working rotating shifts may also experience reduced sleep duration when working night shifts [53], and lower availability of PA resources due to schedules that are asynchronous with the majority of society, resulting in challenges when attempting to establish routines that support PA engagement. Considering that clinical nurses perform a critical service to society on an around-the-clock basis, research is needed to understand how to ease the disruptive nature of clinical and rotating shift work among nurses, in order to enhance their PA behaviour and overall health.

In contrast to total weekly MVPA minutes, our analyses revealed that these same variables (i.e., work role and shift schedule) were not significantly associated with the observed changes in nurses’ average daily step-count. This is promising as it suggests, based on the overall changes observed, that the intervention did have some impact for all nurses regardless of the work situation, and that promoting light PA may be a viable alternative particularly for nurses who find it challenging to engage in recommended levels of weekly MVPA. Average daily step-count reflects all intensities of PA, including light activities that transcend the full spectrum of PA domains (i.e., transportation, housework, occupational and leisure activities). Although the current Canadian and international PA guidelines recommend that people accumulate at least 150 minutes of MVPA per week [41, 42], increasing evidence highlights that reducing sedentary behaviour and increasing light intensity PA may also have important health benefits [54–56], even when accounting for differences in levels of MVPA [57]. It is possible that peo ple w ho face challenges incorporating MVPA into their daily life could benefit by replacing sedentary behaviour with light PA [58]. Thus, research to develop and test in terventions aimed at increasing light PA and reducing sedentary behaviour among nurses, in addition to promoting weekly MVPA, is important.

Contrary to our hypothes es, baseline mood states were not associated with change in total weekly MVPA minutes or change in average daily step-count during the intervention. Our prediction arose from the perspective that more positive affective states, in general, afford people the emotional and cognitive resources required to engage in activities that support personal growth [59, 60], which may include PA. It is also possible that low mood may inhibit engagement in PA behaviour as preoccupation with negative feelings could diminish interest in normally enjoyable activities (e.g., socializing, reading, PA), or prompt more immediate forms of gratification [61]. An examination of the affect-PA literature reveals two possible explanations for our non-significant findings. First, general affective states may interact with individual expectancies regarding PA (i.e., whether a person expects PA to improve or dampen their mood). Previous research has shown that affective states during exercise, which may form the basis of individual expectancies, are strong predictors of future PA behaviour [25, 62, 63]. If this is the case, it is reasonable to assume that individual affective states may vary in their ability to predict individual levels of PA. Second, incidental affective states as a predictor of PA may be time sensitive. Of the few studies that have examined incidental mood states (unrelated to PA) as predictors of PA, significant associations were found between more positive moods and subsequent same day PA [27, 28, 64]. It is possible that POMS, employed as a measure of incidental mood at baseline in the present study, is too global to be a significant predictor of change in PA during a 6-week intervention. The relationship between affect and change in PA during an intervention remains a research question that requires a more nuanced approach and recruitment of a sample that has a greater range of affective state scores.

### Limitations

Our findings should be interpreted in light of the study limitations. Participants were self-selected; nurses who were more motivated to become physically active, or who were healthier and valued PA may have been more likely to volunteer for the study, potentially inflating the effects of the intervention. Second, only observations of PA levels at baseline and during the intervention were considered; follow-up data would be required to determine the long-term effects of the intervention. Third, the study was conducted at a single tertiary care cardiovascular treatment center, and the unique characteristics of this workplace may limit the generalizability of our findings to nurses working in various departments at other hospitals. Larger, multi-center trials of interventions are warranted to further examine the role of shift work and clinical responsibilities on PA behaviour change in nurses. Fourth, this study did not include a comparison or control group. Finally, due to limitations in sample size, all predictors were tested in separate conditional growth models. Comparisons about the relative influence of significant findings should be made with caution.

## Conclusion

Nurses are an important part of the healthcare system, yet through the performance of their duties many sacrifice their own health. Regular PA has been shown to offset some of the negative health consequences associated with nursing, but PA levels among nurses remain low [3, 14–16], and interventions to promote PA behaviour among nurses are scarce [21]. Our study contributes to the literature demonstrating that a simple intervention can lead to increases in total weekly MVPA minutes and average daily step-count among nurses. Our findings highlight that nurses are a diverse population and that differences in the demands of their jobs can have implications of the effectiveness of PA promotion efforts. It is important to account for work-related characteristics and to involve nurses when developing, implementing, and evaluating interventions that can lead to longer-lasting increases in PA levels to enhance mental and physical health in a critically important population.

### Applying research to occupational health practice

A secondary analysis of a three-arm randomized controlled trial was performed to explore whether work-related characteristics and general mood states predicted changes in MVPA and step-count among 70 nurses participating in a web-based worksite PA challenge. When compared to nurses working fixed schedules and performing a combination of clinical, managerial and research duties, those working rotating shifts and performing only clinical work showed smaller improvements in MVPA. These findings suggest that work-related characteristics impact nurses’ respons es to a PA intervention delivered in the workplace, and highlight the need to collaborate with nurses when designing and implementing PA interventions in order to better understand and overcome common work-related barriers to PA (i.e., schedules, work roles).

## Supplementary Information

**Addition al file 1.**

## Data Availability

Researchers may request access to a de-identified database containing work-related characteristics, general mood states, and outcome variables (i.e., total weekly moderate-to-vigorous intensity physical activity and average daily step-count) used for analysis; however, access is contingent on Institutional Review Board approval.

## Abbreviations

-2LL: -2 Restricted Log Likelihood
AIC: Aikaike’s Information Criterion
BIC: Bayesian Information Criterion
MVPA: Moderate-to-vigorous intensity physical activity
PA: Physical activity
POMS: Profile of Mood States
RCT: Randomized controlled trial
REML: Restricted Maximum Likelihood
